# Poor population recovery of lake Washington Chinook Salmon (*Oncorhynchus tshawytscha*) in Pacific Northwestern USA may be associated with disease caused by *Ceratonova shasta* in an urban-influenced watershed

**DOI:** 10.1007/s00436-026-08679-1

**Published:** 2026-04-15

**Authors:** Jan Lovy, Sophie A. Hall, Carl O. Ostberg, Justin B. Greer, Dorothy M. Chase, Genevieve M. A. Kent, Timothy J. Kuzan, Carla M. Conway

**Affiliations:** 1 Western Fisheries Research Center, U.S. Geological Survey, Seattle, WA 98115 USA; 2 Pacific States Marine Fisheries Commission, Portland, OR 97219 USA; 3 Washington Department of Fish and Wildlife, Olympia, Washington 98501 USA; 4 Present address: Department of Public and Ecosystem Health, Cornell University, College of Veterinary Medicine, Ithaca, NY 14853 USA

**Keywords:** *Ceratomyxa shasta*, Ceratomyxosis, Enteronecrosis, Fish disease, Climate change, Anthropogenic

## Abstract

Understanding the effects of anthropogenic changes on fish disease is vital for supporting mitigative actions for fisheries population recovery. The Lake Washington watershed in the Pacific Northwestern United States is influenced by high urban development and climate change, compromising the sustainability of anadromous salmon. We investigated *Ceratonova shasta*, a myxozoan parasite, in Chinook Salmon *Oncorhynchus tshawytscha* to determine the impacts of *C. shasta*-induced enteronecrosis during salmon freshwater migrations within this watershed. All sampled adult Chinook Salmon returning to an enhancement hatchery to spawn were infected with genotype I *C. shasta* with a high prevalence of severe enteritis and enteronecrosis. Parasite loads and associated pathology were more severe in adult fish succumbing to prespawn mortality than in fish that spawned. Natural prespawn mortality of Chinook Salmon in the Cedar River was also associated with high *C. shasta* loads. Sentinel exposure studies with Chinook Salmon juveniles in the spring and fall demonstrated high levels of *C. shasta* genotype II actinospores in the Lake Washington Ship Canal, a human engineered canal that salmon must traverse in their freshwater migrations from the Puget Sound. The sentinel juvenile Chinook Salmon succumbed to clinical disease and high mortality from genotype II *C. shasta*, suggesting that these stocks have high susceptibility to this genotype. The results show that both genotypes I and II pose disease risks to adult and juvenile stages of Chinook Salmon in the watershed, and that the Lake Washington Ship Canal could be a *C. shasta* ‘hot spot’ for genotype II that may increase disease risk for juveniles during smolt emigration.

## Introduction

Chinook Salmon *Oncorhynchus tshawytscha* are an iconic species in Pacific Northwestern North America, being culturally and socio-economically important species for Indigenous communities (Garibaldi and Turner 2004; Nesbitt and Moore 2016) and supporting valuable commercial fisheries (Pacific Salmon Foundation 2025). They are also vital in aquatic ecosystems, being a critical prey species for the southern resident killer whale *Orcinus orca* (Linnaeus, 1758) in the Puget Sound (Stewart et al. 2021; Williams et al. 2011). Puget Sound Chinook Salmon have been listed as threatened by the U.S. Endangered Species Act (ESA) since 1999 with a moderate risk of extinction (Ford 2022). Historically, adult Chinook Salmon returning to spawn in Puget Sound tributaries numbered nearly 700,000, though anthropogenic stressors are believed to have reduced that population by over 90% (WRIA8 Salmon Recovery Council 2017). This is not unique to this area, as most Chinook Salmon populations throughout Pacific Northwestern North America have been in continued decline (Atlas et al. 2023).

The Lake Washington/Cedar/Sammamish watershed, from here referred to as the Lake Washington watershed, is located in western Washington State, USA. The watershed supports natural and hatchery origin anadromous salmon, including Sockeye Salmon *Oncorhynchus nerka*, Coho Salmon *O. kisutch*, and Chinook salmon *O. tshawytscha* (Walbaum 1792), which have been in decline since at least the 1980 s (Fresh 1994). Lake Washington, amid the greater Seattle metropolitan area, is the state’s second largest natural lake, and has undergone heavy urbanization that has largely transformed the limnology and natural flow of the lake (Chrzastowski 1983). The construction of the Lake Washington Ship Canal (LWSC) in 1916, connecting the Puget Sound with Lake Washington, mainly for shipping passage, had a major influence on the watershed. The LWSC construction resulted in a lake elevation drop of about 2.7 m, which dried and severed the connection of the Black River in the southern part of the lake, the historical natural lake outflow, making the LWSC the only entry and exit routes for salmon between the watershed and the Puget Sound (Chrzastowski 1983; Edmondson 1994; Fresh 1994; Urgenson et al. 2021). Along with direct impacts of urbanization on the aquatic ecosystem, Lake Washington has experienced a documented warming water temperature trend since 1963, with the most influence in the epilimnion (Arhonditsis et al. 2004). With the LWSC fed by waters from the Lake Washington epilimnion, salmonids are exposed to sub-lethal water temperatures, approaching lethally warm waters during their migration through the canal, with Chinook salmon particularly vulnerable due to their migration timing (Urgenson et al. 2021). With urbanization and climate-associated habitat degradation believed to be important drivers for population declines, habitat restoration efforts may be necessary to recover salmon in this environment (Fresh and Lucchetti 2020).

Prespawn mortality, when fish arrive on their spawning grounds and die prior to successfully spawning, is believed to be an important contributor to population declines, as is documented in Chinook Salmon populations associated with warm water temperatures in the Willamette River and Columbia River basins (Bowerman et al. 2018, 2021). Exposure to warm water influences pathogen susceptibility and disease, which contributes to reduced survival and prespawn mortality, whereas access to cool water can reduce this mortality, as shown in the Willamette River (Benda et al. 2015). With the severity of infectious diseases in salmon influenced and exacerbated by warming water temperatures (Bruneaux et al. 2017; Kocan et al. 2009; Marcos‐López et al. 2010; Ros et al. 2021), disease will likely limit survival and recovery of salmon.

A major disease of concern in the U.S. Pacific Northwest is caused by the parasite *Ceratonova shasta* (Noble, 1950) (syn. *Ceratomyxa shasta*), which is highly influenced by temperature and has been associated with significant salmon mortality in multiple impaired river systems (Foott 2025; Hallett et al. 2012; Homel and Alexander 2022; Ray et al. 2015, 2012). *Ceratonova shasta* is a cnidarian myxozoan parasite with a complex life cycle requiring alternating hosts, including the annelid *Manayunkia occidentalis* and salmonids, in which actinospores and myxospores are formed in these hosts, respectively (Atkinson et al. 2020; Bartholomew et al. 1997). In salmonids, the parasite causes disease primarily in the intestine, causing enteritis and enteronecrosis, abdominal distension, and systemic spread to other organs (Bartholomew et al. 1989). Previously, *C. shasta* was documented in the Lake Washington watershed (Stinson et al. 2018; Stocking et al. 2007) with consistent detection of the parasite in eDNA surveys within the watershed (Ostberg et al. 2021). The intent of this study was to better understand the impacts of *C. shasta* in threatened Lake Washington Chinook Salmon. To achieve this, we evaluated post-spawned and prespawn mortality Chinook Salmon for *C. shasta* to determine prevalence and to quantify disease severity. Further, sentinel exposure studies were conducted with juvenile Chinook Salmon in strategic locations throughout the watershed to better understand ‘hot spots’ for *C. shasta* transmission and to determine if certain environments are associated with the parasite. Understanding disease risks in Lake Washington Chinook Salmon can be applied to the broader goal of recovering these populations as they face increasing anthropogenic stressors from continued urbanization.

## Materials and methods

### Study location

**Fig. 1:**
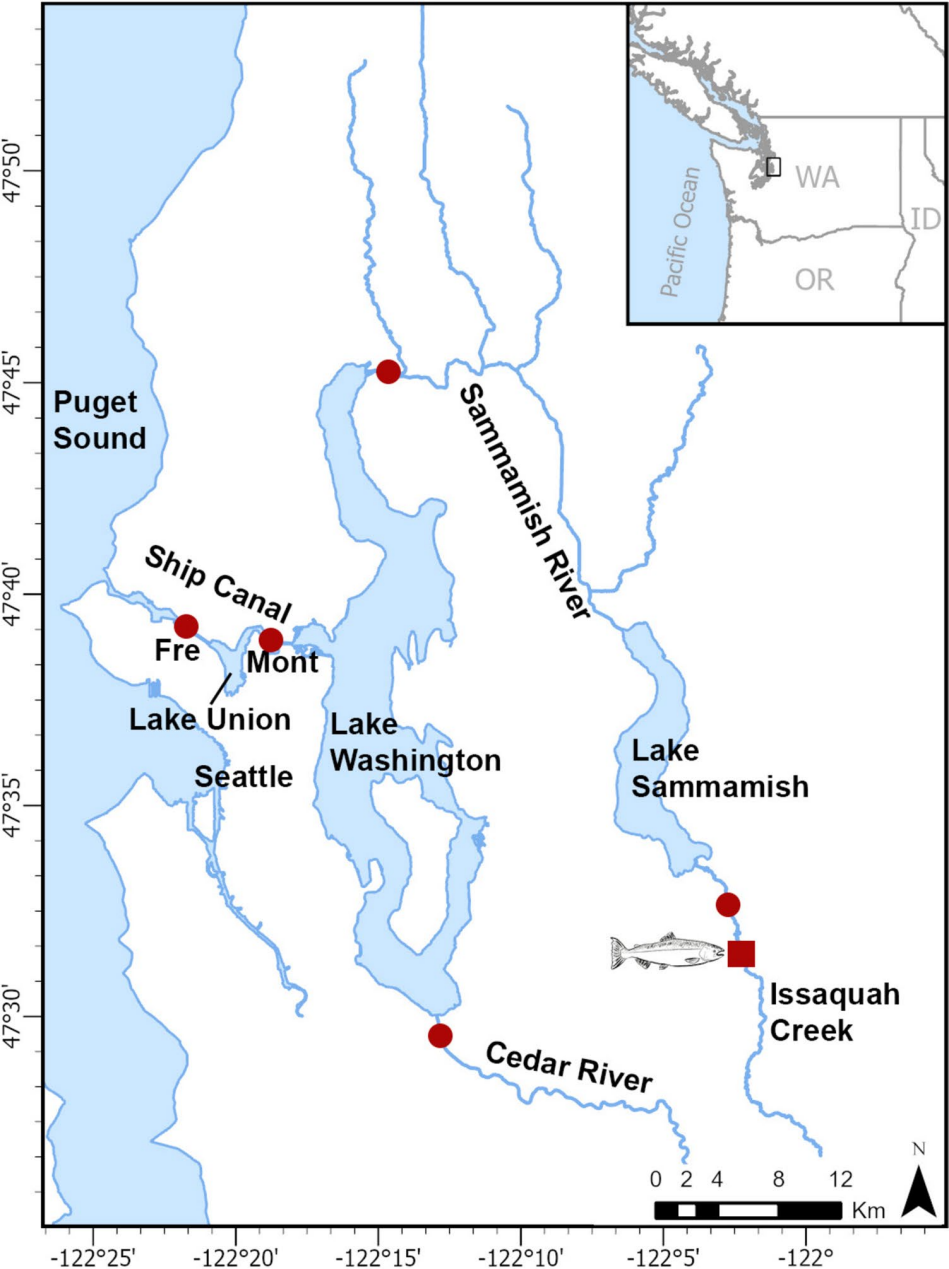
Map showing the Lake Washington watershed with the major lakes and rivers identified. Red circles identify the sentinel caging locations for *Ceratonova shasta* monitoring, including Fremont (Fre), Montlake (Mon), Cedar, Sammamish, and Issaquah Creek. The red box indicates the location of the Issaquah Salmon Hatchery

The Lake Washington watershed is comprised of three lakes and two river systems in western Washington and covers an area of 1792 km^2^ (Fig. 1). This is the most populated watershed in Washington, with 28 cities, including the state’s largest, Seattle, and a growing population estimated at 1.5 million as of 2021 (Urgenson et al. 2021). Lake Washington is connected to the Puget Sound via a human-engineered ship canal, which drains warm epilimnion waters from Lake Washington, through the canal, into the Puget Sound. The Cedar River is the main river system feeding into Lake Washington on the southern end of the lake. A third lake, Lake Sammamish, drains into the Sammamish River and subsequently to the northern end of Lake Washington. On the southern end and draining into Lake Sammamish is Issaquah Creek (Fig. 1).

Natural runs of Chinook Salmon are comprised of two distinct populations, both listed as threatened by the ESA since 1999 (64 FR-14308). One population that spawns in the Cedar River and a second that spawns in Issaquah Creek and tributaries associated with the Sammamish River (Urgenson et al. 2021). The Washington Department of Fish and Wildlife operates the Issaquah Salmon Hatchery (Fig. 1), producing Chinook and Coho Salmon, for release into the watershed.

### Adult chinook salmon

Adult fish were collected in 2024 in conjunction with the Washington Department of Fish and Wildlife and the Muckleshoot Indian Tribe, when spawning adults returned to the watershed from the marine environment. Fish sampling from the Issaquah hatchery included collection of 60 post-spawned fish, lethally sampled on 24-Sep-2024 by euthanizing fish with a pneumatic captive bolt device, and 30 freshly collected fish that experienced prespawn mortality collected between 24-September and 03-October. Spawning surveys of naturally reproducing fish in the Cedar River, conducted by the Muckleshoot Indian Tribe, collected 6 fresh prespawn mortalities between 01-Oct to 08-Oct-2024. Because the Cedar is a small natural population, not associated with a hatchery, limited fish were available and staff only focused on recovering fresh prespawn mortality. No fresh post-spawned fish were available for evaluation.

During tissue collection, internal and external gross lesions were noted, as well as histopathological changes consistent with the presence of a pathogen. For all fish, parallel samples were collected from the posterior intestine and placed into 96% molecular grade ethanol and 10% neutral buffered formalin for molecular screening for *C. shasta* and histopathology, respectively. The same tissue samples were collected from fish that experienced prespawn mortality, with additional organs samples including liver, kidney, and spleen fixed in 10% neutral buffered formalin for histopathological examination. *Ceratonova shasta* genotyping was done from the intestine of 20 post-spawned salmon from the Issaquah Salmon Hatchery and from 11 salmon that succumbed to prespawn mortality, including 5 from the Issaquah Salmon Hatchery and 6 from Cedar River. Molecular and histopathological methods are further described below.

### Juvenile salmon sentinel exposure studies

In 2024, sentinel cages with Chinook Salmon were set up to better understand parasites and other pathogens that juvenile and adult Chinook Salmon encounter during their freshwater migrations in the watershed. A major goal was to determine spatial and temporal risks for infection with *C. shasta*. In 2024, cages were set up during two seasons, spring (29-May to 12-June-2024) and fall (24-Sep to 08-Oct-2024), to correspond with times of Chinook Salmon juvenile emigration (spring) and adult Chinook Salmon spawning migrations (fall). The fall period corresponded with the end of the adult migration because water temperatures were too warm (> 21 °C) in the LWSC to safely hold those fish earlier at those sites. The sentinel fish cages were deployed at the earliest date when temperatures were consistently below 21 °C.

### Fish source

Eyed eggs were sourced from the Issaquah Salmon Hatchery to ensure that the caged fish used for sentinel studies matched the origin of fish found within the watershed. Eyed eggs were transferred to the wet laboratory at the U.S. Geological Survey (USGS) Western Fisheries Research Center in Seattle, Washington. Eggs were first treated with iodophor (1% Argentyne solution) for 10 min and then transferred to disinfected Heath trays for egg incubation. Once hatched fish had mostly absorbed their yolk, ‘buttoned-up,’ they were transferred from Heath trays to flow-through circular tanks and fed daily at 3% body weight (Bio-Oregon, Longview, WA). Water source in the wet laboratory facility throughout the fish holding and rearing period was pathogen-free, filtered, and UV-treated water from Lake Washington. Fish were held with strict temperature control between 8 and 12 °C during egg incubation and fish rearing. Prior to sentinel-exposures in select locations, field water temperatures were measured 1-week prior to cage deployment, and tank temperatures were adjusted no more than 1 °C per day to be within 2 °C of the field water temperatures. Juvenile fish were 3–5 g in weight when they were deployed in sentinel fish cages in both seasons. The mean total length of fish used in spring was 72.1 mm, standard error (SE) = 0.36 (n = 249) and 76.5 mm, SE = 0.63 (n = 139). Juvenile fish were transported via sealed heavy-duty bags with wet laboratory water and compressed O_2_.

### Fish sentinel-exposure procedure

Aluminum cages, 46 cm circumference × 78 cm length, total volume of 0.127 cubic meters with a 0.6 cm mesh size, were used for live sentinel-exposure of juvenile Chinook Salmon. Cages were submerged at least 1 m below the water surface, either suspended in the water column or on hard substrate on the river bottom. Each cage contained between 77 and 118 juvenile fish, for a cage density that was under 3 kg fish/m^3^. In 2024, one fish cage was deployed in each location for a duration of 14 days, with visual checks on the fish and supplemental feeding at 3% body weight at 5- and 10-days post-cage deployment. Temperature monitors (HOBO 64 K Pendant Light and Temperature Logger) were attached to each cage to monitor water temperature every hour for the 14-day duration. To avoid excess thermal stress, cages were not deployed if temperatures at sites exceeded 21 °C.

### Fish sentinel-exposure locations and time periods

In 2024, exposure was done in the spring and fall seasons in four locations, including the Montlake and Fremont sites in the LWSC, one site in the lower Cedar River, and one site in the lower Sammamish River (Fig. 1). These locations were selected based on the presumed habitat preference (lotic environments) for the annelid host *Manayunkia occidentalis*, which occur within the Chinook Salmon migration routes. In 2025, an additional sentinel-exposure site was tested in Issaquah Creek downstream of the salmon hatchery only in the late summer, corresponding with the beginning of Chinook Salmon arrival in Issaquah Creek and at the hatchery. The goal of this site was to determine if Issaquah Creek downstream of the hatchery is an area where fish may be infected with *C. shasta*. Fish were strategically caged directly prior to most of the Chinook Salmon arriving and were only exposed for a 4-day period (18-Aug to 22-Aug) to minimize disruption of migrating salmon in this smaller waterbody.

### Post-cage monitoring of fish

Fish were removed from cages and transferred via aerated coolers to the USGS Western Fisheries Research Center wet laboratory. From the spring-caged fish, 10 fish from each site were euthanized with an overdose of buffered MS-222 (200 mg/L) to screen gill tissue for initial *C. shasta* infection and genotyping. Gills were dissected and fixed into molecular-grade 96% ethanol until further processing for end-point PCR and Sanger sequencing. The remaining live fish were gradually acclimated to match their tank holding temperatures, 15 °C for the fish caged in the spring and 18 °C for fish caged in the fall. All fish from a single cage were put in a single tank. Total fish numbers are summarized in Table 1. Acclimation included gradual addition of wet laboratory water to coolers over a period of 3 h to match the tank water. Fish were held in flow-through circular tanks (volume 3.42 m^3^) on pathogen-free (filtered and UV-treated) water and monitored daily for up to 35 days for mortality. One tank of 60 control fish, which were not subject to sentinel-exposure and held on the same water, were monitored during the same period for each season. Dead fish were examined for disease signs. When skin or gill lesions were observed, skin/gill scrapings were prepared for microscopic wet mount examination. Intestinal wet mounts were prepared by collection of fluid from the vent with a 1 µl bacterial loop and examined with a microscope for *C. shasta* spores and other parasites. Dead fish were labelled and stored at −80 °C until further processing for *C. shasta* molecular testing using quantitative real-time PCR (qPCR). Genotyping of *C. shasta* from intestine of sentinel fish was conducted from 5 different fish for each exposure site during each season, except for instances when *C. shasta* was detected in fewer fish, in which all the available positive samples were genotyped. During post-exposure mortality monitoring, if moribund fish were observed then they were euthanized with an overdose of buffered MS-222 (200 mg/L) and fixed in 10% neutral buffered formalin for histology. Whole fish were placed in the fixative after cutting the operculum and incising the body cavity to allow adequate formalin penetration of organs. Following 48 h in neutral-buffered formalin (NBF), samples were transferred to 70% ethanol until further histology processing. Organs examined included the intestine, liver, kidney, spleen, and gills. In fall-caged fish from Cedar River, 10 previously frozen mortality fish from the period when kidney cell necrosis was noted in histology were tested for infectious hematopoietic necrosis virus (IHNV) using a viral cell culture assay. IHNV screening was done because this virus is known to cause kidney hematopoietic cell necrosis. Briefly, two 5-fish pools of kidney and spleen homogenates were screened using the epithelioma papulosum cyprini (EPC) cell line incubated at 15 °C, following previously published methods (Meyers 2009).

**Table 1:** Summary of 2024 Chinook Salmon *Oncorhynchus tshawytscha* sentinel exposures in spring and fall

2024 Sentinel Exposures			
Location	Criteria	Spring*	Fall
Fremont	Mort/Total fish; cumulative mort	87/108; 89%	96/97; 99%
	PSE gills collected; tested for *Cs*	10; 10	NA
	*Cs* Pos. Morts/Morts tested for *Cs* (qPCR)	21/21	35/35
	*Cs* Pos. Morts/Mort screened for *Cs* (histo)	12/13	4/4
	*Cs* Pos. Survivors/Survivors tested for *Cs* (qPCR)	1/1	0/1
Montlake	Mort/Total fish; cumulative mort	76/118; 70%	86/99; 87%
	PSE gills collected; tested for *Cs*	10; 10	NA
	*Cs* Pos. Morts/Morts tested for *Cs* (qPCR)	20/20	35/35
	*Cs* Pos. Morts/Mort screened for *Cs* (histo)	4/9	4/4
	*Cs* Pos. Survivors/Survivors tested for *Cs* (qPCR)	10/10	10/10
Sammamish River	Mort/Total fish; cumulative mort	40/96; 47%	13/97; 13%
	PSE gills collected; tested for *Cs*	10; 0	NA
	*Cs* Pos. Morts/Morts tested for *Cs* (qPCR)	6/20	3/12
	*Cs* Pos. Morts/Mort screened for *Cs* (histo)	1/4	0/1
	*Cs* Pos. Survivors/Survivors tested for *Cs* (qPCR)	0/10	2/10
Cedar River	Mort/Total fish; cumulative mort	8/80; 11%	38/101; 38%
	PSE gills collected; tested for *Cs*	10; 0	NA
	*Cs* Pos. Morts/Morts tested for *Cs* (qPCR)	5/8	2/20
	*Cs* Pos. Morts/Mort screened for *Cs* (histo)	0/0	0/11
	*Cs* Pos. Survivors/Survivors tested for *Cs* (qPCR)	6/10	1/10

Total mortality (mort) from total number of fish caged with % cumulative mortality reported

^*^Cumulative mortality accounted for an initial lethal sampling of 10 fish post-sentinel exposure (PSE) to screen gills for *Ceratonova shasta* (*Cs*). Only gills from Fremont and Montlake were screened for *C. shasta*. Total numbers of fish intestinal samples from mortalities screened for *C. shasta* by quantitative real-time PCR (qPCR) and histology (histo), and survivors screened for *C. shasta* from intestine by qPCR are reported; not applicable (NA)

### Histopathology

Tissue samples were processed for histology, including dehydration through a series of increasing ethanol concentrations to pure ethanol. Samples were cleared in Clear-Rite 3™ (Thermo Fisher Scientific), permeated and embedded in paraffin wax, and sectioned at a 4 µm thickness. Sections were mounted on glass slides, stained with hematoxylin and eosin, cover-slipped, and examined with a microscope (BX41 Olympus) with a mounted digital camera (4 K-CMOS). Tissue samples were screened for pathological anomalies blinded to study groups. To quantify *C. shasta* induced intestinal pathology, a scoring system related to lesion severity was adapted from Bartholomew et al. (2004). Briefly, scores of 0–5 with increasing numbers reflecting more severe intestinal pathology, were assigned based on the following criteria: (0) *C. shasta* associated pathology was not detected; (1) trophozoites detected with limited or absent inflammation; (2) mild lesions comprised of multiple discrete inflammatory foci surrounding organisms with no mucosal damage or necrosis; (3) moderate lesions comprised of multifocal to diffuse inflammation and necrotic foci with minimal involvement of the intestinal mucosa; (4) moderate-severe lesions consisting of multifocal to diffuse enteritis with severe necrosis of the intestinal mucosa; (5) severe lesions consisting of diffuse transmural inflammation and necrosis with inflammation and myxozoan trophozoites replacing most of the intestinal tissue, with no intact intestinal mucosa remaining. This intestinal pathology scoring was used for field collected adult fish and the juvenile fish used for sentinel exposures.

### Molecular screening and genotyping ceratonova shasta from tissues

#### Quantitative real-time PCR for C. Shasta

Intestine samples were thawed, and a < 25 mg piece of tissue was transferred into a 2 ml centrifuge tube for extraction of DNA using the Qiagen DNEasy blood and tissue kit, according to manufacturer’s directions. qPCR was used to quantify the relative tissue load of *C. shasta* according to Hallett and Bartholomew (2006) with minor modifications. Briefly, a 20 µl reaction volume was used containing 900 nM of each primer, 250 nM probe (Integrated DNA Technologies), 10 µl TaqPath ProAmp Master Mix (Thermo Fisher Scientific), 3.78 µl molecular-grade water, and 5 µl of sample DNA template or molecular-grade water in controls. qPCR was performed on a ViiA 7 Real-Time PCR System (Thermo Fisher Scientific) following the thermocycler parameters described by Hallett and Bartholomew (2006). For each run, samples were run in triplicate alongside positive controls, *C. shasta* Ultramer DNA Oligos (Integrated DNA Technologies), and negative controls, molecular grade water in place of sample DNA template. Transcript copies of *C. shasta* DNA/mg of tissue were determined using a four-point standard curve ranging from 5 × 10^3^ to 5 × 10^6^ copies of the *C. shasta* Ultramer DNA Oligos.

Prior to qPCR, all samples were screened for PCR inhibition using TaqMan Exogenous Internal Positive Control Reagents (IPC) (Thermo Fisher Scientific). An approximately 12.5 µl reaction volume was used containing 6 µl TaqPath ProAmp Master Mix, 1.2 µl 10X Exo IPC Mix, 0.24 µl 50X Exo IPC DNA, and 5 µl of sample DNA template or additional molecular-grade water. Reactions were performed on a ViiA 7 Real-Time PCR System, following the same thermocycler program (Hallett and Bartholomew 2006).

#### Genotype designation by end-point PCR and Sanger sequencing

*Ceratonova shasta* genotype was determined from subsamples of parasite detections in field collected adult Chinook salmon, from gills of juvenile salmon directly after spring sentinel exposure from Montlake and Fremont sites, and from intestines of juvenile sentinel salmon that died during the wet laboratory holding period. PCR amplification of the partial small subunit and the internal transcribed spacer-1 (ITS-1) of the rDNA was conducted as previously described by Atkinson et al. (2018). Briefly, a 50 µl reaction volume was used, containing 25 µl GoTaq G2 Green Master Mix (Promega), 200 nM of each primer (Integrated DNA Technologies), 18 µl molecular-grade water, and 5 µl of sample DNA template or additional molecular-grade water. PCR was performed on a SimpliAmp Thermal Cycler (Thermo Fisher Scientific) following cycle conditions as described by Atkinson et al. (2018). Negative (no template, molecular-grade water) controls were included in each PCR. PCR amplicons were visualized on a 1.5% TBE agarose gel stained with GelRed Nucleic Acid Stain 10000X Water (Merck). PCR products were cleaned using ExoSAP-IT (Thermo Fisher Scientific) following the manufacturer’s instructions. Sequencing reactions were prepared with the cleaned and diluted PCR product and 10 µM of the forward primer (Cs1479F). Sanger sequencing was done by Azenta (Seattle, Washington). Sequences were aligned in BioEdit (Hall 1999; version 7.2.5), and genotype was determined by presence of ATC repeats within the ITS-1, as previously described (Atkinson and Bartholomew 2010; Atkinson et al. 2018).

### Statistical analysis

The relationship between qPCR copies and histology scores were fit using a linear model in R for descriptive purposes only. To assess significant differences in *C. shasta* copy number between pre-spawn mortality and post spawned fish from the Issaquah Hatchery, a Shapiro–Wilk test was first used to investigate normality of the data. Data were determined to not be normally distributed (p < 0.05), and significance was therefore evaluated using the Wilcoxon rank-sum test. Similarly, histopathology scores of pre-spawn and post-spawn mortalities were not normally distributed, and statistical differences were assessed using the Wilcoxon rank-sum test. Generation of Kaplan–Meier survival curves for Chinook Salmon following the sentinel-exposure studies was performed using the survdiff package in R (Therneau 2026). To assess significant differences in mortality across sites, log-rank test p-values were assessed for each individual site comparison, followed by multiple testing correction with Benjamini–Hochberg false discovery correction.

## Results

The underlying data reported below are available in a data release by Hall et al. (2026).

### Adult chinook salmon

#### Issaquah salmon hatchery spawned fish

**Fig. 2:**
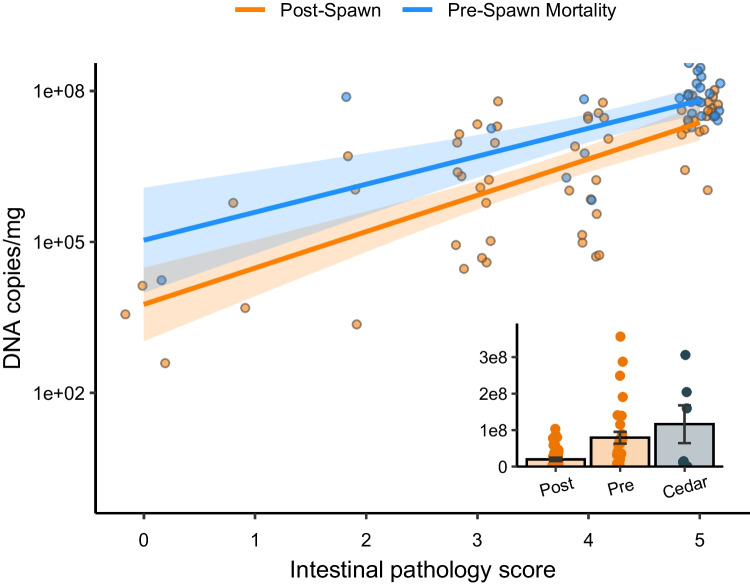
Relationship between intestinal pathology score and DNA copies of *Ceratonova shasta* in spawned fish (Post-Spawn; orange line; linear model, R^2^ = 0.72) and prespawn mortality (blue line; linear model, R^2^ = 0.71) from the Issaquah hatchery. Shaded regions represent 95% confidence intervals for each linear model. Inset: Parasite DNA copies/mg of tissue (y-axis) in fish collected from the Issaquah Salmon Hatchery after successful spawning (post, n = 60), prespawn mortality (Pre, n = 30), and from prespawn mortality from the Cedar River (Cedar, n = 6). Boxplot lines represent the median, hinges correspond to the first and third quartiles, and whiskers are 1.5 times the interquartile range

Gross examinations of the 60 spawned fish indicated that 14/60 had focal necrotizing yellow pigmented lesions in the gill, presumptively identified as columnaris caused by *Flavobacterium columnare*, and 9/60 had small numbers of gill copepods (under 10), presumptively identified as *Salmincola californiensis*. Lower intestines of 59/60 fish had intraluminal hemorrhage. Histopathology detected *C. shasta* in 56/60 fish (Fig. 2) with variable severity based on intestinal histopathology scoring; 35/60 were scored 4 or 5, 24/60 were 3 or under, and 1 fish could not be evaluated due to autolysis of tissue. Molecular testing by qPCR detected *C. shasta* in all 60 samples with a mean of 20 × 10^6^ copies/mg, with high variability (SE = 3.4 × 10^6^) (Fig. 2). A subsample of 20 of these positive samples demonstrated that 18/20 were purely genotype I and 2/20 samples were a mix of genotypes I and II.

#### Issaquah salmon hatchery prespawn mortality

According to counts from Washington Department of Fish and Wildlife, prespawn mortality occurred in 40% of Chinook Salmon at the Issaquah Salmon Hatchery in 2024. Gross evaluation of 30 prespawn mortalities showed that 10/30 fish had focal necrotizing yellow pigmented lesions in gill, presumptively caused by columnaris. 15/30 fish had internal petechial hemorrhage in the viscera and/or infarct of the iris causing the eye to appear blue, suggestive of a bacterial infection. In prespawn Chinook Salmon mortalities sampled 11-Sep-2024, 2/6 cultures taken on TSA media were confirmed as *Aeromonas salmonicida* by conventional PCR using a previously published assay (O’Brien et al. 1994). 26/30 fish had hemorrhage and/or ulcers present in the lower intestine.

In histopathology, three samples could not be evaluated due to extensive autolysis preventing accurate histopathology scoring for *C. shasta*. In the remainder, *C. shasta* was detected in 26/27 samples, with 20/27 having a score of 5, 4/27 with a score of 4, and two samples scoring a 2 and a 3. (Fig. 2). Heavy infections (intestinal score of 5) were also associated with systemic invasion of *C. shasta* trophozoites, most severe in liver, followed by kidney, and then spleen. In 21/30 fish, systemic findings of bacterial aggregations in liver, spleen, and/or kidney were presumptively identified as furunculosis (*A. salmonicida*) based on the bacterial aggregations associated with cellular necrosis and absence of an immune response, typical for furunculosis. Of the 26 fish with *C. shasta*, all but 4 fish were co-infected with furunculosis. Molecular testing for *C. shasta* by qPCR detected the parasite in all 30 samples with a mean of 79.2 × 10^6^ DNA copies/mg with high variability (SE = 16.1 × 10^6^). Genotyping a subsample of 5 positive *C. shasta* intestinal samples showed that all samples were genotype I. When histopathology and DNA copies/mg by qPCR were compared for descriptive purposes by linear regression, histopathology scoring generally correlated with DNA copy number (Fig. 2).

#### Comparison of Issaquah post and prespawn mortality

Comparison of *C. shasta* load by qPCR between the post-spawned fish and prespawn mortality showed that prespawning mortality fish were associated with higher loads of *C. shasta* compared to post-spawned fish (p-value = 1.1 × 10^–5^, Fig. 2 inset). Prespawn mortality fish were also associated with significantly higher histopathology scores (p = 9.6 × 10^–4^, mean score = 4.48) compared to post-spawned fish (mean score = 3.66).

#### Cedar river prespawn mortality

Limited numbers (n = 6) of natural Chinook Salmon prespawn mortality were available for evaluation from the Cedar River. In histology, 5/6 fish had heavy and advanced infections with *C. shasta* and mature myxospores were present in the infected tissues. Three fish had *C. shasta*-related intestinal pathology scores of 5. Two intestine samples were too autolyzed for adequate *C. shasta* histopathology scoring. One of these autolyzed samples had heavy numbers of *C. shasta* present in the autolyzed intestine, while another sample had severe *C. shasta* trophozoite invasion in liver, kidney, and spleen, indicating that these fish were infected, despite the inability to accurately score the lesions. One fish did not have *C. shasta* associated lesions in the intestine with a histological score of 0. Molecular testing for *C. shasta* by qPCR demonstrated all 6 samples were positive for the parasite. The one fish that had a *C. shasta* histopathology score of 0 had relatively low *C. shasta* DNA copy numbers (3.2 × 10^4^ DNA copies/mg). The other five fish had high levels of *C. shasta* with a mean of 1.2 × 10^8^ DNA copies/mg (SE = 51.8 × 10^6^) (Fig. 2). Genotyping of *C. shasta* from all six fish showed that they were purely genotype I.

### Juvenile chinook salmon sentinel exposure

#### Spring exposure

**Fig. 3:**
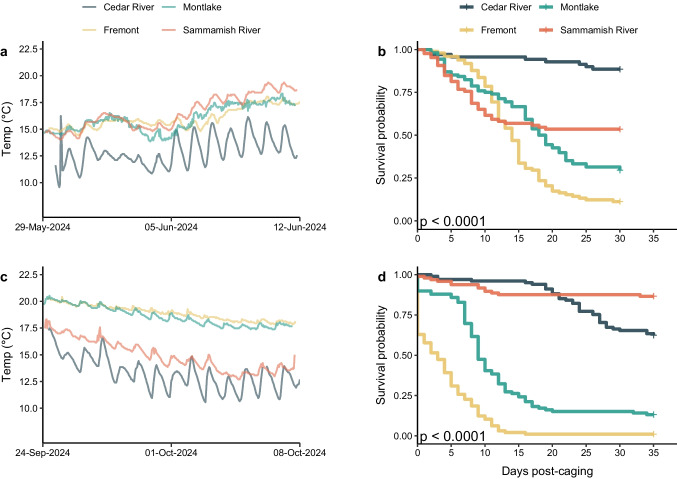
Sentinel-exposure of Chinook Salmon, *Oncorhynchus tshawytscha*, in the spring (**a**, **b**) and the fall (**c**, **d**). Water temperatures during the sentinel caging period of Chinook salmon at four sites in 2024 (**a**, **c**) and the subsequent fish survival probability during wet laboratory holding and monitoring following exposure in four sites (**b**, **d**). For each season, all sites showed statistically significant differences in mortality from all other sites (p < 0.05)

Water temperatures during the 14-day exposure were site specific and ranged between 10 and 19 °C (Fig. 3a). Water temperatures were cooler with higher daily fluctuations in the Cedar River than other sites (Fig. 3a). Fish survival during the exposure period was high with only one dead fish from the cage held in the Sammamish River. Following transfer to the wet laboratory, total cumulative mortality 30 days post-exposure (PE) varied between the sites (Table 1). Kaplan–Meier survival analysis demonstrated that fish from both sites in the LWSC were less likely to survive compared to the other sites, and all sites were statistically different from each other (p < 0.05, Fig. 3b). Details on site specific mortality are below.

**Fig. 4:**
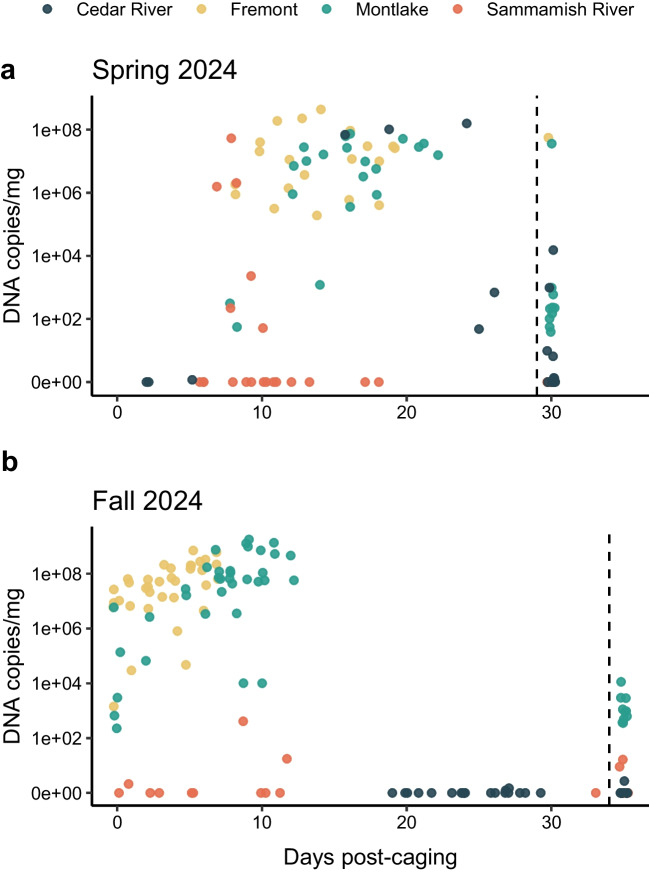
*Ceratonova shasta* DNA copies/mg of intestinal tissue from fish during laboratory monitoring following sentinel-exposure from four sites, including two sites in the Lake Washington Ship Canal (Fremont and Montlake) in the spring (**a**) and fall (**b**). Points to the left of the dotted line are from mortalities and points to the right are from surviving fish at the end of the monitoring period

Cedar River: No gross disease signs were observed in the low mortality from the Cedar River-held fish during the monitoring period and no moribund fish were available for evaluation by histology. All 8 mortalities were positive for *C. shasta* by qPCR with variable parasite loads and 4/10 fish that survived to the end of the study had detectable *C. shasta* (Fig. 4).

Sammamish River: The Sammamish River-held fish experienced mortality during the first 10-days PE, which subsided during the remaining holding period. Clinical signs, including tail and fin rot, were consistent with columnaris, and microscopic wet mounts in those fish showed high numbers of bacteria from skin and gills. Bacteria were long rods that formed aggregates in microscopic wet mounts, presumptively identified as *Flavobacterium columnare*. No gross clinical signs of *C. shasta* were noted in any of the fish during the holding period. Histology on 4 moribund fish from Sammamish confirmed external gill bacteria in 2/4 fish and *C. shasta* was confirmed in two fish: one 4-days PE with myxozoan trophozoites in the liver only and the other fish 8 days PE with an intestinal pathology score of 4. Molecular detection of *C. shasta* by qPCR detected *C. shasta* in 6/10 mortalities and in 0/10 fish that survived to the end of the study (Fig. 4).

Montlake- Lake Washington Ship Canal: No infectious pathogens were observed grossly or by histology in the 3 moribund fish collected 4-days PE. *C. shasta* was first detected by histology in one moribund fish 6-days PE, which had an intestinal pathology score of 2. Clinical signs of *C. shasta*, distended abdomens and swollen vents, started at 15-days PE and continued to 26-days PE. In histology, a moribund fish 14-days PE had a *C. shasta* intestinal pathology score of 3 with systemic spread of developmental myxozoan stages to other organs. One moribund fish at day-20 PE had external gill bacteria, presumptively identified as columnaris. Two moribund fish, 22- and 23-days PE had *C. shasta* intestinal pathology scores of 4 and 5, respectively, with systemic spread in the fish at 22-days PE. Molecular testing by qPCR confirmed *C. shasta* in all 20 mortalities tested and low levels of *C. shasta* in the surviving fish sampled on the final day of monitoring (Fig. 4).

**Fig. 5:**
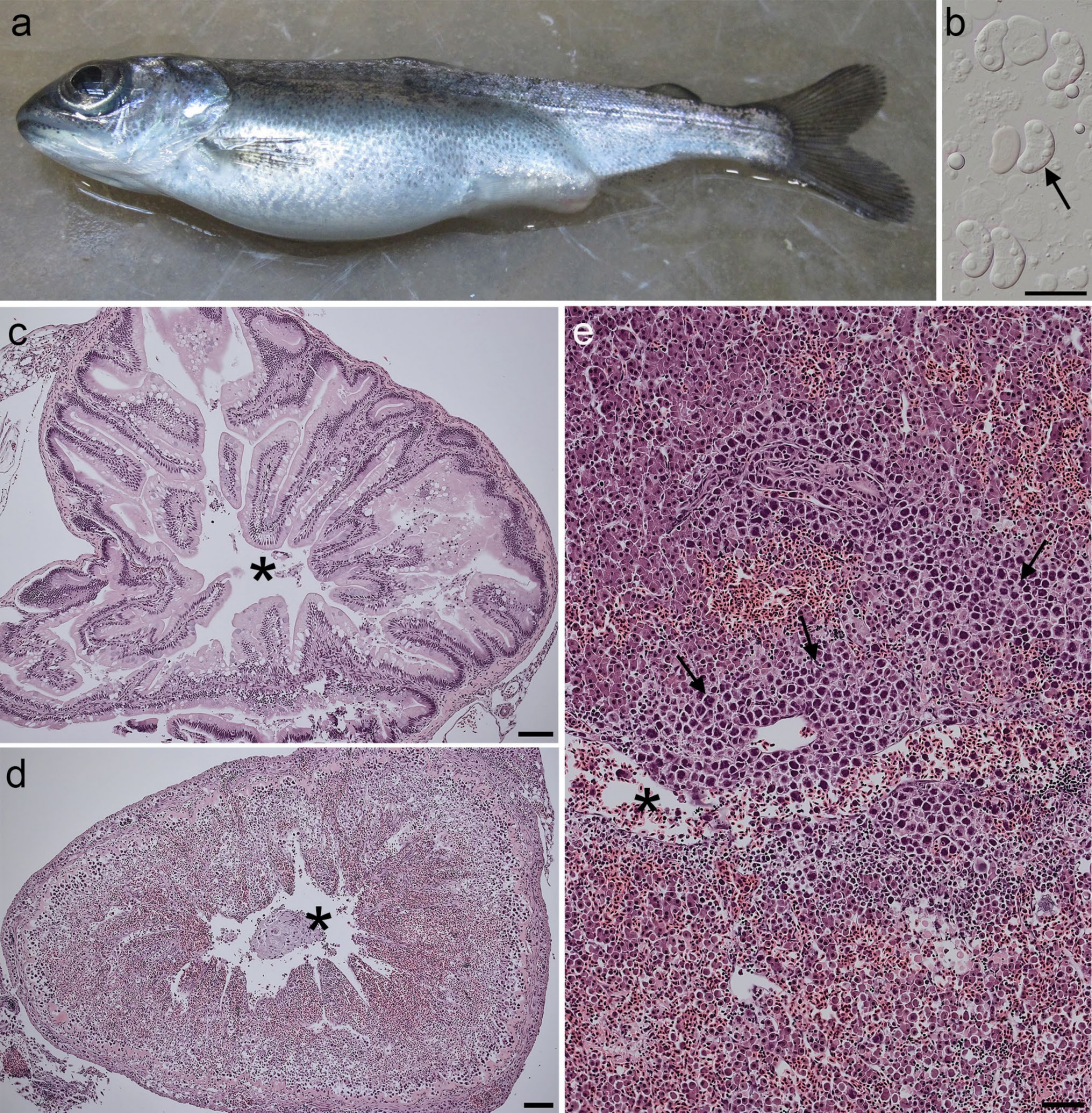
Sentinel juvenile Chinook salmon, *Oncorhynchus tshawytscha*, with *Ceratonova shasta*-induced enteronecrosis related to genotype II during peak mortality from the Lake Washington Ship Canal. (**a**) Severe abdominal distension and a protruding/swollen vent. (**b**) Microscopic wet mount of *Ceratonova shasta* myxospores taken from a vent swab (bar = 20 µm). Histology of an unaffected intestine (**c**) and a severely infected intestine with an intestinal pathology score of 5 (**d**) (* intestinal lumen; bars = 100 µm). Notice in (**d**) the loss of the intestinal mucosa and severe infiltration extending from the lamina propria to the muscularis with myxozoan trophozoites and inflammatory cells. (**e**) Liver with myxozoan trophozoite invasion of tissue (arrows) and blood vessels (*), associated with hemorrhage, inflammation, and displacement of hepatocytes (bar = 50 µm)

Fremont- Lake Washington Ship Canal: Clinical signs of *C. shasta*, including distended abdomens, swollen and occasionally hemorrhaged vents, enteritis, and enteronecrosis with myxospores observed in intestinal wet mounts (Fig. 5), first occurred at 9-days PE. In histology, *C. shasta* was first detected in two moribund fish at 9-days PE with intestinal pathology scores of 5. All moribund fish sampled during the monitoring period 9- to 20-days PE were confirmed infected with *C. shasta* with majority of fish with intestinal pathology scores of 5 (9 fish), followed by 4 (3 fish), and a single fish 20-days PE with an intestinal score of 3. One of the moribund fish had a co-infection with an intestinal flagellate, presumptively identified as *Hexamita salmonis*. No other pathogens were noted grossly or in histology. Molecular screening by qPCR detected *C. shasta* in 20/20 samples from mortality fish (Fig. 4).

*Ceratonova shasta* genotyping: 20 gill samples taken immediately following sentinel caging, 10 from Fremont and 10 from Montlake, demonstrated all gill samples contained purely genotype II *C. shasta*. Gills from the other two sites were not evaluated, since *C. shasta* infections did not develop in the majority of those fish. All *C. shasta* samples evaluated from intestines of PE mortalities (5 from each site, with only 3 genotyped samples from the Sammamish) showed that all 18 samples were purely genotype II.

#### Fall caging

Water temperature in caging sites varied by location and ranged between 11 and 20 °C, with the two sites in the LWSC warmer than both the Cedar and Sammamish River sites (Fig. 3). The Cedar River again showed higher daily temperature fluctuations than the other sites. During the final three days of caging, in-cage mortality occurred mainly in the two LWSC sites, with 42% mortality (36 fish) occurring in Fremont and 10% mortality (10 fish) in Montlake. Sammamish had one in-cage mortality, and the Cedar River did not have any mortality during the caging period.

Following transfer to the wet laboratory, cumulative mortality during the 35-day holding period is summarized in Table 1 and Fig. 3. Kaplan–Meier survival analysis showed that fish in the two sites within the LWSC were less likely to survive compared to the other sites, and all sites are statistically different from each other (p < 0.05, Fig. 3d). Further details on site-specific mortality are below.

Cedar River: Rare mortalities before 19-days PE appeared to be related to mechanical/traumatic injury from caging, secondary bacterial infection, and at least in one fish a systemic fungal infection. In histology, one of two moribund fish collected on the day of transfer to the wet laboratory showed a small focus of epithelial hyperplasia in gill associated with bacteria, which was not further identified. The second fish had no findings in histology. From two moribund fish evaluated in histology 3-days PE, one had no significant findings, and the other had a systemic fungal infection with mats of fungal hyphae present in the kidney, intestine, and spleen associated with cellular necrosis. During the notable increase in mortality between 20- and 35-days PE, a total of 7 moribund fish were evaluated by histology. All 7 fish had high numbers of melanomacrophage centers in kidney and 3 of those fish had diffuse hematopoietic cell necrosis primarily in the anterior and mid-kidney. Additionally, in 4 of those fish eosinophilic globules, likely protein, occurred within the Bowman’s space of glomeruli. Cellular degeneration and high numbers of melanomacrophage centers also occurred in the spleen of 3 fish. No significant findings occurred in any of the other organs. Throughout the monitoring period, no histological signs of *C. shasta* occurred in any of the moribund fish, and intestinal wet mounts of moribund fish did not detect *C. shasta* myxospores. Molecular screening for *C. shasta* by qPCR only detected very light *C. shasta* in 2 mortality samples (< 5 copies/mg) and a single light positive in a survivor fish (12 copies/mg) at the end of the study (Fig. 4; Table 1). Screening 10 fish from this late mortality for infectious hematopoietic necrosis virus using viral cell culture assays were negative for the virus. Based on the absence of granulomatous lesions, as would be seen in bacterial kidney disease (*Renibacterium salmoninarum*), it is unlikely that mortality was related to this. These tests ruled out potential causes for mortality, though a definitive cause for mortality was not determined and may be related to environmental factors rather than pathogens.

Sammamish River: Due to the low level of mortality, only one moribund fish was available for histopathology at 9-days PE. This moribund fish had a systemic fungal infection in kidney, spleen, and intestine with large numbers of fungal hyphae in the organs associated with cellular necrosis. No histologic signs of *C. shasta* associated pathology were noted and no gross signs of *C. shasta* were present in any fish observed during the holding period. Molecular screening for *C. shasta* by qPCR detected low levels in 3/12 intestinal samples from fish that succumbed to mortality, ranging from 8–411 DNA copies/mg tissue and 2/10 very low detections in intestinal samples from fish that survived to the end of the study (9 and 16 DNA copies/mg tissue) (Fig. 4).

Montlake- Lake Washington Ship Canal: Ten mortalities occurred within the cage during the final 3 days of holding. Due to their poor condition, these fish were not necropsied or available for histology. Of these dead fish collected on the final day of caging 5/5 were positive for *C. shasta* by qPCR, ranging from 227 to 5.9 × 10^6^ DNA copies/mg tissue (Fig. 4). During the monitoring period after caging, a notable rise in mortality occurred after 5-days PE with highest mortality occurring between 7- and 13-days PE (55 mortalities). Moribund fish had distended abdomens, swollen vents, and occasional hemorrhage around the vent. Intestinal wet mounts examined microscopically first confirmed mature *C. shasta* myxospores on day-9 PE. Four moribund fish collected between 6- and 10-days PE evaluated for histology all had severe intestinal pathology related to *C. shasta* with histology scores of 5. All 4 fish had systemic spread of the myxozoan developmental stages to liver and spleen, with *C. shasta* trophozoites also noted within the kidney of 2 fish. One of 4 fish also had regionally severe necrosis within the gill associated with the presence of long bacterial rods, presumptively identified as columnaris. Based on qPCR screening of intestinal tissue from 30 mortalities occurring during the holding period, *C. shasta* was detected in all samples (Fig. 4). *Ceratonova shasta* was still detectable in fish that survived to the end of the study 35-days PE, though at relatively low levels, ranging from 373 to 1.1 × 10^4^ DNA copies per mg tissue (Fig. 4).

Fremont- Lake Washington Ship Canal: Of the 36-caging mortalities that were collected on the final day of caging 5/5 were positive for *C. shasta* by qPCR at relatively high levels, ranging from1430 to 2.7 × 10^7^ DNA copies/mg tissue (Fig. 4). Two moribund fish taken on the day of and the day following transfer into the wet laboratory from the field cage were evaluated for histology. One fish sampled the day of transfer had *C. shasta*-associated lesions in the intestine, with an intestinal pathology score of 2, and mild hepatitis associated with myxozoan trophozoites in the liver. The second fish had *C. shasta* associated lesions in the intestine only, with an intestinal pathology score of 4. Two additional moribund fish that died 3-days PE were evaluated by histology, and one had severe intestinal pathology (score of 5) and many myxozoan trophozoites associated with hepatitis, while the other fish had a low-grade infection, with an intestinal pathology score of 1. No other lesions suggestive of other pathogens or diseases were detected by histology. Intestinal wet mounts first detected mature *C. shasta* myxospores 7-days PE. qPCR monitoring of intestinal samples from 30 mortalities throughout the holding period were all positive, at high levels, for *C. shasta* (Fig. 4). Only a single fish remained at the end of the holding period, from which the intestinal sample was negative for *C. shasta* by qPCR.

*Ceratonova shasta* genotyping: Ten intestinal samples, 5 from each of the LWSC sites (Montlake and Fremont) were genotype II *C. shasta*. Because of the low detection level of *C. shasta* from the Sammamish River, only a single sample was successfully genotyped and was confirmed as genotype II. No positive samples were available for genotyping from the Cedar River.

### 2025 caging in Issaquah creek

The temperature ranged between 13 and 18 °C with a mean of 16 °C during the 4-day caging period. Upon transfer of fish to the wet laboratory, within the first 26-days of monitoring only a single mortality occurred 5-days PE. Between 27- and 35-days PE, 20 mortalities occurred. Necropsy and microscopic wet mounts showed these mortalities were associated with external bacteria characterized by clumping long filamentous rods. Fish had pale gills, fin and tail rot, and ulcerating skin lesions. This was presumptively identified as columnaris infection. These fish did not show any gross signs suggestive of *C. shasta,* and all intestinal wet mounts were negative for *C. shasta* myxospores. Considering this is the time range when *C. shasta* would be expected, these 20 mortalities were tested using the *C. shasta* qPCR and 4 out of 20 samples were lightly positive (3; 14; 39; 1055 DNA copies/mg of tissue). Three of these samples were genotyped; 2 samples were confirmed as genotype I, and one sample was genotype II. 10 more mortalities occurred between days 37 to 47 of the monitoring period. Those fish showed the same signs of columnaris infection and intestinal wet mounts were all negative for *C. shasta*.

## Discussion

*Ceratonova shasta* has often been associated with mortality of juvenile fishes in other systems (Homel and Alexander 2022; Lehman et al. 2020). In adult fish, the parasite was found commonly in Chinook Salmon in the Willamette River, Oregon (Kent et al. 2013, 2014), though it is not suspected as the primary cause driving prespawn mortality (Nervino et al. 2024). Our results showed that adult fish succumbing to prespawn mortality at the Issaquah Salmon Hatchery had higher *C. shasta* levels and associated intestinal pathology when compared to spawned fish, suggesting the parasite had a role in prespawn mortality. Co-infections may also exacerbate mortality, as furunculosis was commonly noted in fish suffering from prespawn mortality. It is possible that the intestinal tract damage (enteronecrosis) caused by *C. shasta* may leave salmon more vulnerable to bacterial diseases such as furunculosis, as entry of the bacterium *A. salmonicida* is believed to occur via the digestive tract (Jutfelt et al. 2008). Similarly, co-infections with *Flavobacterium* spp. infections, presumptively columnaris, persistently occurred in adult fish, likely contributing to disease. The heavy *C. shasta* loads in the examined six adult prespawn mortalities associated with severe pathology in the Cedar River was highly notable, as this is a high priority area for restoration, since it provides spawning and rearing areas for the largest natural-origin Chinook Salmon population in the watershed (WRIA 8 Salmon Recovery Council 2017). Considering the low sample size from the Cedar River, future efforts to increase numbers of fish examined could help to understand if this is a common trend. Cedar River Chinook Salmon have a different migration route than the Sammamish population, yet both had heavy *C. shasta* infections. This suggests that infection could happen in overlapping habitats early in migration, such as in the LWSC and/or Lake Washington itself. For both populations, it was not surprising that genotype I *C. shasta* was the predominant genotype found since this is the parasite genotype known to cause disease mainly in Chinook Salmon (Hurst and Bartholomew 2012). Genotype I is considered specific to Chinook Salmon with high virulence, whereas genotype II generally does not cause mortality in enzootic strains of Chinook Salmon (Hurst and Bartholomew 2012). Co-infections with these two genotypes have been reported in Chinook salmon, with genotype I often overtaking and masking genotype II infections in diseased fish because of its increased virulence (Hurst and Bartholomew 2012). This is supported by the findings herein as 2/20 postspawned adult salmon were co-infected with both genotypes I and II.

Identifying areas where *C. shasta* infection occurs in adult fish can help focus efforts on mitigating certain habitats within the watershed. Infection with *C. shasta* is limited to freshwater via actinospore shedding from the freshwater-specific annelid, *Manayunkia occidentalis* (Atkinson et al. 2020; Bartholomew et al. 1997; Pettibone 1953), and thus infection may occur anywhere between entry into the LWSC and the respective spawning grounds. Our fall sentinel caging study, intended to identify *C. shasta* during the time when adults are migrating into the watershed, clearly showed high infection in sentinel fish in the two LWSC sites. Though susceptibility to the parasite is likely higher in adult spawning fish, due to their immunocompromised status, high *C. shasta* associated mortality occurred in the juvenile sentinel fish (see Table 1 and Figs. 3,4). Unexpectedly, *C. shasta* detected in all the caging sites in 2024 were genotype II and not genotype I, which was the predominant genotype found in field-collected adult Chinook Salmon from this study. This suggests that adult fish are infected elsewhere by genotype I. In 2025, a fifth caging site was tested to determine if genotype I infection may occur downstream of the Issaquah Salmon Hatchery, considering that adult salmon with heavy infections are expected to shed mature myxospores downstream into Issaquah Creek. Low *C. shasta* levels of both genotypes I and II were detected by qPCR in only 4/20 sentinel fish, suggesting this may not be an important infection site with either genotype. The shorter caging period (4-days) must also be considered in reference to this low detection of *C. shasta*, as other sites had caging periods of 14-days. Further sentinel caging studies, or molecular monitoring for the parasite from water samples, is necessary in other sites in the watershed to better understand where fish are being infected with genotype I.

An important finding was the apparent high susceptibility of Lake Washington juvenile Chinook Salmon to *C. shasta* genotype II. A host-parasite genotype association has been demonstrated throughout Pacific Northwestern North America, with genotype O occurring in Rainbow/Steelhead Trout *Oncorhynchus mykiss* (Walbaum, 1792), genotype I in Chinook Salmon, and genotype II a generalist found in various salmonid species though most often associated with Coho Salmon (Atkinson and Bartholomew 2010; Bartholomew et al. 2022; Stinson et al. 2018). It was unexpected that genotype II would be so lethal in Chinook Salmon based on the host-parasite genotype associations described in other regions. In the juvenile sentinel exposures from the LWSC, gross clinical signs including severe abdominal distension combined with severe intestinal pathology suggested that *C. shasta* was a primary pathogen responsible for mortality. The histopathological findings in adult fish with genotype I and juvenile fish with genotype II were similar. Severe infections in both life stages were marked by severe transmural inflammation, enteronecrosis with sloughing of the intestinal mucosa, and parasite spread to other internal organs.

The Chinook Salmon in the Lake Washington basin have been reported to belong to three main spawning groups (stocks), including Issaquah Creek, Bear Creek, and Cedar River, though they are generally managed as two stocks- a Sammamish (northern Lake Washington) and a Cedar River stock (Berge et al. 2006; Warheit and Bettles 2005). Despite this stock separation, Lake Washington Chinook Salmon exhibit high genetic homology, which may be explained by the common origin of these fish from the Green River (Warheit and Bettles 2005). The high genetic similarity suggests that it is unlikely for the stocks to be distinguished enough to drive susceptibility differences to *C. shasta*, though this has not been tested. The juvenile fish used within the sentinel cages herein were the same stock/strain that is used for hatchery supplementation into the Lake Washington watershed; thus, this susceptibility to *C. shasta* genotype II reflects that of the Chinook Salmon population within the watershed. This suggests that emigrating juvenile Chinook Salmon could face a high risk of infection and mortality from *C. shasta* genotype II, particularly when migrating through the LWSC. This is supported by the spring sentinel caging study, which was timed when juvenile Chinook Salmon were emigrating into the marine environment. Juvenile Chinook Salmon must migrate through the LWSC to reach the marine environment, and we suspect that the longer the time spent in the LWSC will likely increase the risk of disease from *C. shasta*. It is reported that juvenile Chinook Salmon may spend between 3 days and 2 weeks within the LWSC prior to marine emigration (Urgenson et al. 2021), thus the 14-day caging duration herein represents the higher end of the actual time that a juvenile fish would spend in the LWSC. Future field sampling of juvenile Chinook Salmon over a spatial and temporal scale could help to determine infection levels in naturally migrating salmon as they migrate through the watershed, including the LWSC, prior to reaching the marine environment.

The high susceptibility of Lake Washington Chinook Salmon to genotype II follows a pattern suggesting that Chinook Salmon in this system have not co-evolved with *C. shasta*, as resistance to *C. shasta* is common in salmonids within the enzootic range (Bartholomew 1998). In the Klamath River, sympatric juvenile Chinook Salmon, fish that naturally co-occur with the parasite, are highly resistant to genotype II, though allopatric fish, ones from regions in which the parasite and fish host do not co-occur, suffer from mortality, likely because of a lack of host–pathogen coevolution (Hurst and Bartholomew 2012). In addition to our findings here, there was one earlier report of genotype II *C. shasta* associated with mortality from a Chinook Salmon hatchery utilizing water from Lake Washington/LWSC (Stinson et al. 2018). The history of *C. shasta* in the Lake Washington watershed is not fully understood, including how long this parasite may have been present with the host Chinook Salmon population. In 2003–2004, surveillance for *C. shasta* in the Puget Sound showed that the parasite was absent in most river systems, except for a light infection from a fish in the Deschutes River and low prevalence in fish held at the University of Washington Aquaculture Research (UW-ARC) hatchery in Portage Bay, Lake Washington (Stocking et al. 2007). This shows that *C. shasta* has been present in Lake Washington at least as early as 2003, though the parasite was generally absent or rare from the surrounding Puget Sound rivers (Stocking et al. 2007), which could suggest that the parasite was a more recent introduction compared to enzootic rivers, including the Columbia and Klamath River basins (Hoffmaster et al. 1988; Stocking et al. 2006).

The historical absence of *C. shasta* within the Puget Sound rivers, particularly in the Green and Duwamish Rivers (Stocking et al. 2007) where the Lake Washington Chinook Salmon stocks originated, and the high susceptibility of these Chinook Salmon to genotype II suggests a lack of parasite-host co-evolution in this region. This may explain the susceptibility differences in fish within Lake Washington compared to the Klamath River basin. This is further supported by work demonstrating that hatchery fall Chinook Salmon from the Columbia River basin were highly resistant to *C. shasta*, whereas hatchery strains derived from coastal rivers were highly susceptible (Zinn et al. 1977). If *C. shasta* is a more recent introduction, then the sympatric Chinook Salmon in Lake Washington may lack resistance to genotype II. Further research directed towards understanding the co-evolution of *C. shasta* and Lake Washington Chinook Salmon could better explain parasite-associated mortality in this system. Hatchery-supplemented stocks have been demonstrated to exacerbate *C. shasta* by increasing infectious levels of the parasite in the Klamath River (Robinson et al. 2020). It is possible that managers may select *C. shasta* resistant stocks for supplementation in the watershed as a mitigation strategy to avoid disease. However, introducing genetically different salmon stocks to a watershed may not be possible as hatchery supplementation programs strive to maintain genetic integrity of endemic fish stocks. Further, it is unknown if a different genetic stock will be more susceptible to other endemic pathogens in the watershed. It will be interesting to understand if the current stocks gain resistance to *C. shasta* over time as may occur in other systems that have a long history of parasite/host co-evolution.

Based on the sentinel caging studies here, the time from exposure to development of *C. shasta*-induced disease by genotype II in Chinook Salmon depended on the site, season, and temperature. Exact disease progression cannot be resolved since fish were continuously exposed over a 14-day caging period. Infections were more severe and had an earlier onset in fall-caged fish when compared to the spring. In the spring, *C. shasta*-induced disease occurred between 9 and 26 days following their 14-day field exposure, with peak disease occurring about 6 days earlier in Fremont compared to Montlake. In the fall, peak *C. shasta*-induced disease and mortality occurred directly after caging to about 13-days PE, with peak mortality in Fremont being several days earlier than Montlake. These differences in timing and severity are likely explained by temperature and actinospore concentrations that fish were exposed to. The warmer caging temperatures and holding fish at 18 °C following sentinel-exposure in the fall may explain the increased severity of disease and earlier disease onset compared to the spring-caged fish. Further, the low infections in the Cedar River are likely influenced by temperature, as this site had cooler water temperatures when compared to the other sites. This temperature influence on disease progression and severity has been well described in the Klamath River (Ray et al. 2012). Waterborne actinospore concentrations increase with water temperatures up to 18 °C, whereas these actinospore concentrations are reduced once water temperature reaches 23 °C due to spore degradation (Ray and Bartholomew 2013). Site specific differences may explain differences in actinospore concentrations, since temperatures did not vary considerably between Montlake and Fremont within the LWSC, though disease onset was earlier in Fremont compared to Montlake. If high densities of the annelid *M. occidentalis* and infectious actinospores are present in the LWSC, then it may be expected to see this higher severity in Fremont, as it is located downstream of Montlake and could have a higher accumulation of actinospores from upstream locations in the LWSC. Density of actinospores has been demonstrated to influence mortality in Coho and Chinook Salmon, though time-to-death did not appear to be influenced by actinospore density of genotype I in Chinook Salmon (Hallett et al. 2012). The time-to-death of Chinook Salmon did vary in the study herein, though it should be noted that these infections were related to genotype II and thus cannot be directly compared to genotype I infections in the Klamath basin. In the Klamath, actinospore density of genotype II did influence the disease onset and time to death in Coho Salmon (Hallett et al. 2012), so perhaps that is more comparable to the findings of genotype II in Chinook Salmon herein. It was notable to see high *C. shasta*-induced disease in fall-caged fish in the LWSC, whereas parasite prevalence and infection severity trended lower in the other river sites. In the Klamath River, actinospore density was reported to peak in the early summer followed by a reduction in the fall, with higher parasite-induced mortality rates at temperatures greater than 15 °C (Hallett et al. 2012). This raises questions regarding *C. shasta* prevalence in the LWSC and if certain factors, such as temperature and habitat, contribute to its persistence at high levels compared to natural river systems.

The high persistence of infectious actinospores in the LWSC, as shown in the fall sentinel exposures, may be influenced by multiple factors, with one being a high abundance of the annelid definitive host *M. occidentalis*. Understanding the preferred habitat of *M. occidentalis* in natural river environments is important to better understand if the LWSC may contain suitable habitat for heavy colonization of the annelid host for *C. shasta*. *Manayunkia occidentalis*, previously incorrectly reported as the closely related *M. speciosa* (Atkinson et al. 2020), are small annelids, 3 mm in length that reside within and attach to substrate via a tube constructed of fine particles and mucoid secretions (Atkinson et al. 2020; Pettibone 1953). These annelids are tolerant of a wide range of environmental conditions and feed via a branchial plume of tentacles used for capturing food from water currents (Poe and Stefan 1974). These annelids are associated with slow water currents and do not tolerate fast-moving or stagnant water (Mackie and Qadri 1971; Stocking and Bartholomew 2007). Tolerance to water flow is dependent on attachment substrate (Malakauskas et al. 2013) with the preferred habitat being substrates that contain silt and fine benthic organic matter in slow flows of 0.02–0.05 m/s, with annelid densities increasing as flow velocity decreases (Stocking and Bartholomew 2007). Increased flows may be tolerated when the organisms may be partially sheltered, for example, they are frequently associated with *Cladophora* sp. that form thick algal mats containing silt and fine benthic organic matter (Stocking and Bartholomew 2007). High water velocity following floods may be an important natural mechanism in rivers to control abundance of *M. occidentalis*, as increased flows will dislodge and remove these annelids (Stocking and Bartholomew 2007). As such, in environments like the Klamath River, where *C. shasta* accounts for annual juvenile Chinook Salmon mortality, manipulation of water flow via dam release to dislodge these organisms has been a method to control *C. shasta* (Alexander et al. 2014; Bartholomew et al. 2023). The LWSC is a 10.8 km long excavated and dredged engineered canal with its banks heavily armored with cement, docks, and piers (Chrzastowski 1983; Urgenson et al. 2021). The low water flow, controlled via the Ballard Locks, and the engineered substrate may create stable conditions suitable for high colonization with this annelid. Water temperature is another important consideration in the LWSC, as water temperature influences parasite development in *M. occidentalis* (Ray et al. 2015) and the salmonid fish host (Ray et al. 2012). The LWSC water is from the epilimnion of Lake Washington, and thus, these are the warmest water temperatures that migrating Chinook Salmon face within the watershed (Urgenson et al. 2021). This may be an important factor driving infections with *C. shasta*. Future efforts in understanding the LWSC habitat in relation to *M. occidentalis* abundance may identify methods to reduce this host and ultimately the incidence of *C. shasta*-induced disease in Chinook Salmon.

The dense annelid populations alone cannot explain high concentrations of *C. shasta* actinospores, as that habitat must overlap with salmonid hosts shedding mature myxospores to ensure transmission and parasite establishment (Atkinson et al. 2020; Bartholomew et al. 1997). Considering that the LWSC is the only entry and exit for adult/juvenile salmonids, all salmonids within the system will be present within the LWSC at some point, though their timing may not necessarily coincide with myxospore shedding. With our sentinel exposure studies, mature genotype II myxospores were first detected in Chinook Salmon from both spring and fall at around 7–9 days following a 14-day sentinel-exposure, thus it may take roughly 3 weeks for mature myxospores to develop following initial infection. Considering that juvenile Chinook Salmon only persist in the LWSC for up to 2 weeks, it is unlikely for them to both become infected and shed myxospores during their transit period through the LWSC, though it is possible for them to become infected elsewhere and shed myxospores while transiting the LWSC. Natural and hatchery supplemented stocks of Coho Salmon also occur in this watershed. Considering that genotype II is often associated with Coho Salmon (Atkinson et al. 2018; Stinson et al. 2018), it is probable that they contribute to the transmission and cycling of genotype II within the watershed. Though detailed freshwater life history of juvenile Coho Salmon within the Lake Washington basin is not available, high mortality has been documented in hatchery-released coho during their freshwater migration prior to entering the marine environment (Muckleshoot Indian Tribe 2016). Other salmonid species that may be present which could be possible hosts for genotype II include Cutthroat Trout *Oncorhynchus clarkii* (Richardson 1836) and Rainbow Trout. Though Steelhead Trout *Oncorhynchus mykiss* were historically present, these are believed to be functionally extirpated following declines since the 1980 s (Urgenson et al. 2021). Understanding *C. shasta* in these trout species would be valuable to better understand if they may serve as important hosts for *C. shasta* genotype II in Lake Washington.

It is possible that spawning adult fish may also contribute to seeding the LWSC environment with genotype II myxospores, despite mainly finding genotype I in adults at their spawning grounds. Some adult spawning Chinook Salmon entering the LWSC delay at Ballard Locks for a period of up to 37 days as they are thought to adjust to abrupt temperature and salinity changes between the Puget Sound and the LWSC (Urgenson et al. 2021). During this time, they are exposed to effluent from the LWSC, and thus fish that delay at Ballard Locks could become infected and shed myxospores by the time they migrate through the LWSC. Even if adult fish do shed myxospores during their transit through the LWSC, then it would not be for long, since once they enter the LWSC they move through it in under 1 day (Urgenson et al. 2021). Further, it is thought that myxospore shedding from adult fish occurs post-mortem on spawning grounds (Kent et al. 2014), and if that is the case here, then myxospore shedding is unlikely to occur in the LWSC. If adult Chinook Salmon are infected by genotype II upon entry to the ship canal, then it is likely that they are infected with genotype I elsewhere in the watershed, which would then overtake the genotype II infections. This could be tested by sampling adult fish and testing them for *C. shasta* early in their spawning migration to determine if they are infected and shedding genotype II myxospores.

Our findings highlight the presence of two *C. shasta* genotypes in the Lake Washington basin that may contribute to disease in juvenile and adult Chinook salmon. This freshwater habitat is compromised by thermal stress and habitat alterations related to urbanization and climate change. Efforts to identify and improve habitats within the basin have become a priority, as further anthropogenic stressors could make the environment unsustainable for salmonids (Fresh and Lucchetti 2020; Urgenson et al. 2021). Future habitat mitigation in the watershed may consider methods to reduce *C. shasta* to avoid parasite-induced mortality in the population. In mitigating the Lake Washington habitat, many lessons may be learned from control strategies employed in the historically dammed Klamath River (Bartholomew et al. 2023) and how they may apply to control strategies in the Lake Washington watershed. Different drivers are likely at play, i.e., a dammed natural river in the Klamath, compared to limnological changes from construction of the LWSC and habitat alteration from urbanization in the Lake Washington watershed. Despite these differences, mitigation strategies to control the parasite will likely focus on similar principles to reduce biotic and abiotic parasite transmission factors (Bartholomew et al. 2022), including thermal refuge/temperature manipulation (Chiaramonte et al. 2016; Ray et al. 2015, 2012), water flow manipulation (Alexander et al. 2014; Ray et al. 2015; Ray and Bartholomew 2013; Stocking and Bartholomew 2007), and methods to remove/reduce annelids in critical habitats to reduce disease risks for Chinook salmon.

## Data Availability

All data underlying the conclusions of this manuscript can be found in the following data release: Hall, S.A., J. Lovy, C.O. Ostberg, J.B. Greer, D.M. Chase, G.M.A. Kent, T.J. Kuzan, and C.M. Conway. 2026. Field surveillance and sentinel exposures to detect *Ceratonova shasta* in Chinook Salmon from the Lake Washington watershed. U.S. Geological Survey data release. 10.5066/P13ZJB5J

## References

[CR1] Alexander JD, Hallett SL, Stocking RW, Xue L, Bartholomew JL (2014) Host and parasite populations after a ten year flood: *Manayunkia speciosa* and *Ceratonova* (syn *Ceratomyxa*) *shasta* in the Klamath River. Northwest Sci 88(3):219–233

[CR2] Arhonditsis GB, Brett MT, DeGasperi CL, Schindler DE (2004) Effects of climatic variability on the thermal properties of Lake Washington. Limnol Oceanogr 49(1):256–270

[CR3] Atkinson SD, Bartholomew JL (2010) Disparate infection patterns of *Ceratomyxa shasta* (Myxozoa) in rainbow trout (*Oncorhynchus mykiss*) and Chinook salmon (*Oncorhynchus tshawytscha*) correlate with internal transcribed spacer-1 sequence variation in the parasite. Int J Parasitol 40(5):599–604 19895812 10.1016/j.ijpara.2009.10.010

[CR5] Atkinson SD, Bartholomew JL, Rouse GW (2020) The invertebrate host of salmonid fish parasites *Ceratonova shasta* and *Parvicapsula minibicornis* (Cnidaria: Myxozoa), is a novel fabriciid annelid, *Manayunkia occidentalis* sp. nov.(Sabellida: Fabriciidae). Zootaxa 4751(2):310–320-310-320 10.11646/zootaxa.4751.2.6 32230420

[CR4] Atkinson SD, Hallett SL, Bartholomew JL (2018) Genotyping of individual *Ceratonova shasta* (Cnidaria: Myxosporea) myxospores reveals intra-spore ITS-1 variation and invalidates the distinction of genotypes II and III. Parasitology 145(12):1588–1593 29580305 10.1017/S0031182018000422

[CR6] Atlas WI et al (2023) Trends in Chinook salmon spawner abundance and total run size highlight linkages between life history, geography and decline. Fish Fish 24(4):595–617

[CR7] Bartholomew JL (1998) Host resistance to infection by the myxosporean parasite *Ceratomyxa shasta*: a review. J Aquat Anim Health 10(2):112–120

[CR11] Bartholomew JL, Alexander JD, Alvarez J, Atkinson SD, Belchik M et al (2023) Deconstructing dams and disease: predictions for salmon disease risk following Klamath River dam removals. Front Ecol Evol 11:1245967

[CR10] Bartholomew JL, Alexander JD, Hallett SL, Alama-Bermejo G, Atkinson SD (2022) *Ceratonova shasta*: a cnidarian parasite of annelids and salmonids. Parasitology 149:1862–1875 36081219 10.1017/S0031182022001275 PMC11010528

[CR66] Bartholomew JL, Ray E, Torell B, Whipple MJ, Heidel JR (2004) Monitoring *Ceratomyxa shasta* infection during a hatchery rearing cycle: comparison of molecular, serological and histological methods. Dis Aquat Org 62:85–92 10.3354/dao062085 15648834

[CR8] Bartholomew JL, Rohovec J, Fryer JL (1989) *Ceratomyxa shasta*, a myxosporean parasite of salmonids. Fish Disease Leaflet 80. U.S. Fish and Wildlife Service, Washington, D.C., p 8

[CR9] Bartholomew JL, Whipple M, Stevens D, Fryer JL (1997) The life cycle of *Ceratomyxa shasta*, a myxosporean parasite of salmonids, requires a freshwater polychaete as an alternate host. J Parasitol 83(5):859–868 9379291

[CR12] Benda SE, Naughton GP, Caudill CC, Kent ML, Schreck CB (2015) Cool, pathogen-free refuge lowers pathogen-associated prespawn mortality of Willamette River Chinook Salmon. Trans Am Fish Soc 144(6):1159–1172

[CR13] Berge H, Hammer M, Foley S (2006) Timing, abundance, and population characteristics of spawning chinook salmon in the Cedar. Sammamish Watershed. Technical report by the King County Conservation District. https://your.kingcounty.gov/dnrp/library/2006/kcr1960.pdf. Accessed 13 Jan 2026

[CR15] Bowerman TE, Keefer ML, Caudill CC (2021) Elevated stream temperature, origin, and individual size influence Chinook salmon prespawn mortality across the Columbia River Basin. Fish Res 237:105874

[CR14] Bowerman T, Roumasset A, Keefer ML, Sharpe CS, Caudill CC (2018) Prespawn mortality of female Chinook salmon increases with water temperature and percent hatchery origin. Trans Am Fish Soc 147(1):31–42

[CR16] Bruneaux M, Visse M, Gross R, Pukk L, Saks L, Vasemägi A (2017) Parasite infection and decreased thermal tolerance: impact of proliferative kidney disease on a wild salmonid fish in the context of climate change. Funct Ecol 31(1):216–226

[CR17] Chiaramonte LV, Ray RA, Corum RA, Soto T, Hallett SL, Bartholomew JL (2016) Klamath River thermal refuge provides juvenile salmon reduced exposure to the parasite *Ceratonova shasta*. Trans Am Fish Soc 145(4):810–820

[CR18] Chrzastowski MJ (1983) Historical changes to Lake Washington and route of the Lake Washington Ship Canal, King County, Washington. Open File Report. U.S. Geological Survey. 81–1182. 10.3133/ofr811182

[CR19] Edmondson W (1994) Sixty years of Lake Washington: a curriculum vitae. Lake Reserv Manage 10(2):75–84

[CR20] Foott JS (2025) Ceratonova shasta infection in Sacramento River Chinook salmon. California Fish and Wildlife Scientific Journal 111(2): 10.51492/cfwj.111.7

[CR21] Ford MJ (2022) Biological viability assessment update for Pacific salmon and steelhead listed under the Endangered Species Act: Pacific Northwest. U.S. Department of Commerce, NOAA Technical Memorandum NMFS-NWFSC-171. 10.25923/kq2n-ke70

[CR22] Fresh KL (1994) Lake Washington fish: a historical perspective. Lake Reserv Manage 9(1):148–151

[CR23] Fresh KL, Lucchetti G (2020) Protecting and restoring the habitats of anadromous salmonids in the Lake Washington Watershed, an urbanizing ecosystem Sustainable Fisheries Management. CRC Press, p 525–544

[CR24] Garibaldi A, Turner N (2004) Cultural keystone species: implications for ecological conservation and restoration. Ecol Soc 9(3):1

[CR27] Hallett SL, Bartholomew JL (2006) Application of a real-time PCR assay to detect and quantify the myxozoan parasite *Ceratomyxa shasta* in river water samples. Dis Aquat Organ 71(2):109–118 16956058 10.3354/dao071109

[CR28] Hallett SL et al (2012) Density of the waterborne parasite *Ceratomyxa shasta* and its biological effects on salmon. Appl Environ Microbiol 78(10):3724–3731 22407689 10.1128/AEM.07801-11 PMC3346369

[CR26] Hall SA, Lovy J, Ostberg CO, Greer JB, Chase DM, Kent GMA, Kuzan TJ, and Conway CM (2026) Field surveillance and sentinel exposures to detect Ceratonova shasta in Chinook Salmon from the Lake Washington watershed. U.S. Geological Survey data release. 10.5066/P13ZJB5J

[CR25] Hall TA (1999) BioEdit: a user-friendly biological sequence alignment editor and analysis program for Windows 95/98/NT. Nucleic Acids Symp Ser 41:95–98

[CR29] Hoffmaster J, Sanders J, Rohovec J, Fryer JL, Stevens D (1988) Geographic distribution of the myxosporean parasite, *Ceratomyxa shasta* Noble, 1950, in the Columbia River basin, USA. J Fish Dis. 10.1111/j.1365-2761.1988.tb00528.x

[CR30] Homel K, Jd A (2022) Spatiotemporal distribution of *Ceratonova shasta* in the lower Columbia River Basin and effects of exposure on survival of juvenile chum salmon *Oncorhynchus keta*. PLoS One 17(8):e0273438 36018896 10.1371/journal.pone.0273438 PMC9417023

[CR31] Hurst C, Bartholomew J (2012) *Ceratomyxa shasta* genotypes cause differential mortality in their salmonid hosts. J Fish Dis 35(10):725–732 22808922 10.1111/j.1365-2761.2012.01407.x

[CR32] Jutfelt F, Sundh H, Glette J, Mellander L, Thrandur Björnsson B, Sundell K (2008) The involvement of *Aeromonas salmonicida* virulence factors in bacterial translocation across the rainbow trout, *Oncorhynchus mykiss* (Walbaum), intestine. J Fish Dis 31(2):141–151 18234022 10.1111/j.1365-2761.2007.00879.x

[CR33] Kent ML, Benda S, St-Hilaire S, Schreck CB (2013) Sensitivity and specificity of histology for diagnoses of four common pathogens and detection of nontarget pathogens in adult Chinook salmon (*Oncorhynchus tshawytscha*) in fresh water. J Vet Diagn Invest 25(3):341–351 23536613 10.1177/1040638713482124

[CR34] Kent ML, Soderlund K, Thomann E, Schreck CB, Sharpton TJ (2014) Post-mortem sporulation of *Ceratomyxa shasta* (Myxozoa) after death in adult Chinook Salmon. J Parasitol 100(5):679–683 24725089 10.1645/13-490.1

[CR35] Kocan R, Hershberger P, Sanders G, Winton J (2009) Effects of temperature on disease progression and swimming stamina in *Ichthyophonus*-infected rainbow trout, *Oncorhynchus mykiss* (Walbaum). J Fish Dis 32(10):835–843 19570061 10.1111/j.1365-2761.2009.01059.x

[CR36] Lehman BM, Johnson RC, Adkison M, Burgess OT, Connon RE et al (2020) Disease in Central Valley Salmon: status and lessons from other systems. San Francisco Estuary Watershed Sci 18(3):2. https://doi.org/10.15447//sfews.2020v18iss3art2

[CR37] Mackie G, Qadri S (1971) A polychaete, *Manayunkia speciosa*, from the Ottawa River, and its North American distribution. Can J Zool 49(5):780–782

[CR38] Malakauskas DM, Willson SJ, Wilzbach MA, Som NA (2013) Flow variation and substrate type affect dislodgement of the freshwater polychaete, *Manayunkia speciosa*. Freshw Sci 32(3):862–873

[CR39] Marcos-López M, Gale P, Oidtmann B, Peeler E (2010) Assessing the impact of climate change on disease emergence in freshwater fish in the United Kingdom. Transbound Emerg Dis 57(5):293–304 20561287 10.1111/j.1865-1682.2010.01150.x

[CR40] Meyers TR (2009) Fish pathology section laboratory manual. Alaska Department of Fish and Game, Division of Commercial Fisheries. 3rd Edition. Special Publication No. 12. https://www.adfg.alaska.gov/static/fishing/pdfs/research/pathology_labmanual_v3.pdf. Accessed 13 Jan 2026.

[CR41] Muckleshoot Indian Tribe (2016) Monitoring Coho Salmon Smolt Outmigration Survival in Lake Washington. Prepared by the Muckleshoot Indian Tribe Fisheries Division, December 2016 Auburn, Washington. https://www.govlink.org/watersheds/8/reports/pdf/Lake_Washington_Coho_Smolt_Study_2015_Final_Report.pdf. Accessed 13 Jan 2026

[CR42] Nervino S, Polley T, Peterson JT, Schreck CB, Kent ML, Alexander JD (2024) Intestinal lesions and parasites associated with senescence and prespawn mortality in Chinook Salmon (*Oncorhynchus tshawytscha*). J Fish Dis 47(2):e13876 37888803 10.1111/jfd.13876

[CR43] Nesbitt HK, Moore JW (2016) Species and population diversity in Pacific salmon fisheries underpin indigenous food security. J Appl Ecol 53(5):1489–1499

[CR65] Noble ER (1950) On a myxosporidian (protozoan) parasite of California trout. J Parasitol 36:457–460 14795328

[CR44] O’Brien D, Mooney J, Ryan D, Powell E, Hiney M, Smith PR, Powell R (1994) Detection of *Aeromonas salmonicida*, causal agent of furunculosis in salmonid fish, from the tank effluent of hatchery-reared Atlantic salmon smolts. Appl Environ Microbiol 60(10):3874–3877 7527205 10.1128/aem.60.10.3874-3877.1994 PMC201900

[CR45] Ostberg CO, Chase DM, Purcell MK (2021) Spatial and temporal survey of waterborne myxozoan parasites in the Lake Sammamish watershed, Washington, from 2019–2020. U.S. Geological Survey data release. 10.5066/P9MGLG1Z

[CR46] Pacific Salmon Foundation (2025) State of Salmon 2025 Report. Pacific Salmon Foundation, Vancouver, BC, Canada. 10.60740/stateofsalmon.2025

[CR47] Pettibone MH (1953) Fresh-water polychaetous annelid, *Manayunkia speciosa* Leidy, from Lake Erie. Biol Bull 105(1):149–153

[CR48] Poe TP, Stefan DC (1974) Several environmental factors influencing the distribution of the freshwater polychaete, *Manayunkia speciosa* Leidy. Chesapeake Sci 15(4):235–237

[CR51] Ray AR, Alexander JD, De Leenheer P, Bartholomew JL (2015) Modeling the effects of climate change on disease severity: a case study of *Ceratonova* (syn *Ceratomyxa*) *shasta* in the Klamath River Myxozoan evolution, ecology and development. Springer, p 363–378

[CR49] Ray AR, Bartholomew JL (2013) Estimation of transmission dynamics of the *Ceratomyxa shasta* actinospore to the salmonid host. Parasitology 140(7):907–916 23506996 10.1017/S0031182013000127

[CR50] Ray AR, Holt RA, Bartholomew JL (2012) Relationship between temperature and *Ceratomyxa shasta*–induced mortality in Klamath River salmonids. J Parasitol 98(3):520–526 22746389 10.1645/JP-GE-2737.1

[CR52] Robinson HE, Alexander JD, Hallett SL, Som NA (2020) Prevalence of infection in hatchery-origin Chinook Salmon correlates with abundance of *Ceratonova shasta* spores: implications for management and disease risk. N Am J Fish Manag 40(4):959–972

[CR53] Ros A et al (2021) Current and projected impacts of the parasite *Tetracapsuloides bryosalmonae* (causative to proliferative kidney disease) on Central European salmonid populations under predicted climate change. Freshw Biol 66(6):1182–1199

[CR54] Stewart J et al (2021) Survival of the fattest: linking body condition to prey availability and survivorship of killer whales. Ecosphere 12(8):e03660

[CR55] Stinson ME, Atkinson SD, Bartholomew JL (2018) Widespread distribution of *Ceratonova shasta* (Cnidaria: Myxosporea) genotypes indicates evolutionary adaptation to its salmonid fish hosts. J Parasitol 104(6):645–650 30142293 10.1645/18-79

[CR56] Stocking RW, Bartholomew JL (2007) Distribution and habitat characteristics of *Manayunkia speciosa* and infection prevalence with the parasite *Ceratomyxa shasta* in the Klamath River, Oregon–California. J Parasitol 93(1):78–88 17436945 10.1645/GE-939R.1

[CR57] Stocking RW, Holt RA, Foott JS, Bartholomew JL (2006) Spatial and temporal occurrence of the salmonid parasite *Ceratomyxa shasta* in the Oregon-California Klamath River basin. J Aquat Anim Health 18(3):194–202

[CR58] Stocking RW, Lorz HV, Holt RA, Bartholomew JL (2007) Surveillance for *Ceratomyxa shasta* in the Puget Sound watershed, Washington. J Aquat Anim Health 19(2):116–120 18201052 10.1577/H06-029.1

[CR59] Therneau T (2026) A Package for Survival Analysis in R. R Package Version 3.8–6, https://CRAN.R-project.org/package=survival.

[CR60] Urgenson L, Kubo J, DeGasperi C (2021) Synthesis of Best Available Science: Temperature and Dissolved Oxygen Conditions in the Lake Washington Ship Canal and Impacts on Salmon. Prepared for the Lake Washington, Cedar, Sammamish Watershed (WRIA 8) Salmon Recovery Council. https://www.govlink.org/watersheds/8/pdf/2021TempDOShipCanalScienceRpt_6_8_21.pdf. Accessed 14 Jan 2026

[CR61] Warheit KI, Bettles CM (2005) Genetic Characterization of Chinook Salmon (Onchorhynchus tshawytscha) populations within Watershed Resource Inventory Area 8 (WRIA 8), Washington State. Genetics Laboratory, Washington Department of Fish and Wildlife, Olympia, WA. https://www.govlink.org/watersheds/8/reports/warheit&bettlesfinal_sept2005.pdf. Accessed 14 Jan 2026

[CR62] Williams R, Krkosek M, Ashe E, Branch TA, Clark S et al (2011) Competing conservation objectives for predators and prey: estimating killer whale prey requirements for Chinook salmon. PLoS ONE 6(11):e26738 22096495 10.1371/journal.pone.0026738 PMC3212518

[CR63] WRIA 8 Salmon Recovery Council. (2017) Lake Washington/Cedar/ Sammamish Watershed Chinook Salmon Conservation Plan 10-year Update. Water Resource Inventory Area (WRIA) 8, Seattle, WA. http://www.govlink.org/watersheds/8/ reports/plan-update.aspx. Accessed 14 Jan 2026

[CR64] Zinn J, Johnson K, Sanders J, Fryer J (1977) Susceptibility of salmonid species and hatchery strains of Chinook salmon (*Oncorhynchus tshawytscha*) to infections by *Ceratomyxa shasta*. J Fish Res Board Can 34(7):933–936

